# Cow’s Milk Bioactive Molecules in the Regulation of Glucose Homeostasis in Human and Animal Studies

**DOI:** 10.3390/foods13172837

**Published:** 2024-09-06

**Authors:** Emad Yuzbashian, Emily Berg, Stepheny C. de Campos Zani, Catherine B. Chan

**Affiliations:** 1 Department of Agriculture, Food and Nutritional Science, University of Alberta, Edmonton, AB T6G 2P5, Canada; yuzbashi@ualberta.ca; 2 Department of Physiology, University of Alberta, Edmonton, AB T6G 2H7, Canada; eberg@ualberta.ca (E.B.); zani@ualberta.ca (S.C.d.C.Z.)

**Keywords:** dairy, milk, insulin resistance, type 2 diabetes, glucose, insulin, incretin, inflammation, gut microbiome

## Abstract

Obesity disrupts glucose metabolism, leading to insulin resistance (IR) and cardiometabolic diseases. Consumption of cow’s milk and other dairy products may influence glucose metabolism. Within the complex matrix of cow’s milk, various carbohydrates, lipids, and peptides act as bioactive molecules to alter human metabolism. Here, we summarize data from human studies and rodent experiments illustrating how these bioactive molecules regulate insulin and glucose homeostasis, supplemented with in vitro studies of the mechanisms behind their effects. Bioactive carbohydrates, including lactose, galactose, and oligosaccharides, generally reduce hyperglycemia, possibly by preventing gut microbiota dysbiosis. Milk-derived lipids of the milk fat globular membrane improve activation of insulin signaling pathways in animal trials but seem to have little impact on glycemia in human studies. However, other lipids produced by ruminants, including polar lipids, odd-chain, trans-, and branched-chain fatty acids, produce neutral or contradictory effects on glucose metabolism. Bioactive peptides derived from whey and casein may exert their effects both directly through their insulinotropic effects or renin-angiotensin-aldosterone system inhibition and indirectly by the regulation of incretin hormones. Overall, the results bolster many observational studies in humans and suggest that cow’s milk intake reduces the risk of, and can perhaps be used in treating, metabolic disorders. However, the mechanisms of action for most bioactive compounds in milk are still largely undiscovered.

## 1. Introduction

The regulation of glucose homeostasis within the body depends mainly on the tight regulation of insulin secretion from pancreatic β-cells and its actions on peripheral tissues, including muscle, adipose tissue, and liver [1]. However, impaired secretion of insulin or the existence of insulin resistance (IR) in the peripheral tissues results in chronic hyperglycemia, which leads to the manifestation of impaired glucose homeostasis and the development of metabolic diseases, such as type 2 diabetes mellitus (T2DM). The progression of T2DM and its morbidities causes a significant societal and individual financial burden and is a leading cause of premature mortality and reduced life expectancy [2]. The prevalence of T2DM has risen globally in the last two decades, largely due to obesity, which is attributed to overnutrition and lack of physical activity [3]. Adherence to a healthy diet plan is one of the primary strategies for managing obesity and normalizing glucose homeostasis. Of the dietary components currently under investigation for reducing the risk for T2DM, dairy products have received considerable attention as inducing improvements in insulin sensitivity and a favorable metabolic profile [4,5,6].

Milk is a highly consumed dairy product, but whether it promotes glucose homeostasis or contributes to the risk of T2DM remains a debated topic. A meta-analysis of 35 observational studies including nearly 400,000 participants recently concluded that the highest consumption of both total dairy and milk was associated with a decreased risk of incident metabolic syndrome (comprising pre-diabetes and T2DM) of 20% and 17%, respectively [7]. In a meta-analysis of cohort studies, each 200 g/day increment in consumption of total dairy and milk was associated with a 25% and 7% reduced likelihood of becoming overweight and obese, respectively. A neutral association was found between milk consumption and the risk of T2DM [8]. However, some individual studies revealed adverse [9,10] or null [11,12] effects that may depend on the baseline glycemic state of individuals included in the study. Overall, these epidemiological studies suggest that milk has functional properties that contribute to the regulation of glucose homeostasis, such as increasing insulin sensitivity, enhancing insulin-secreting capacity, or improving glucose metabolism within the liver [13,14,15] but are limited by study designs that cannot determine causation and have inherent residual confounding.

Furthermore, the idea that milk is beneficial is challenged by evidence that milk has strong insulin secretory activity [16], which may cause acute hyperinsulinemia and exacerbate IR [17,18]. On the other hand, by acutely raising insulin, euglycemia may be re-established more rapidly to reduce the demand on the pancreas for insulin [19]. It is essential to consider milk’s complexity, as it contains numerous bioactive compounds that might influence glucose regulation in different directions individually; thus, their resultant effects could be beneficial. Well-designed clinical trials are needed to help resolve these discrepant results, some of which are described in the following sections.

Cow’s milk is a mixture that predominantly consists of water (about 87%). Fats, carbohydrates, protein, vitamins and minerals constitute the remaining 13%, thus making the milk nutritionally dense [20]. Notably, milk contains many bioactive molecules that may elicit metabolic impacts beyond its basic nutritional value [21,22]. The macronutrients are a rich source of diverse bioactive molecules, such as lactose, galactose, and milk oligosaccharides from carbohydrates, the milk fat globule membrane (MFGM), even-, odd- and branched-chain fatty acids, and milk-specific trans fatty acids, along with protein hydrolysates and bioactive peptides derived from milk proteins [23]. Milk also contains other ingredients with bioactivities, including the milk microbiota and microRNAs. As the focus of this review is on the bioactive molecules derived from macronutrients, we refer the reader to several recent reviews covering the milk microbiota and microRNAs [24,25,26,27]. The first objective of the present review is to outline the effects of bioactive compounds found in milk carbohydrates, lipids, and proteins in relevant human clinical trials. The second objective is to discuss how milk bioactive molecules improve glucose regulation using data from animal and cell culture models. This narrative review synthesizes relevant literature from PubMed, Scopus, and Google Scholar, using the appropriate terms and keywords, focusing on human studies, animal experiments, and in vitro studies up to 2024 related to cow’s milk bioactive molecules and glucose homeostasis.

## 2. Carbohydrates

Lactose is the most abundant carbohydrate in cow’s milk but varies in amount due to many factors, for example the cow’s diet and lactation stage [28]. Cow’s milk also contains small amounts of lactose-derived products such as lactulose, lactitol, lactobionic acid, and galactooligosaccharides [29,30]. Milk has a low glycemic index (GI) and low glycemic load (GL), meaning that its consumption yields a lower blood glucose response compared with an equivalent dose of glucose, which is attributed to slower hydrolysis and absorption of lactose [31,32]. The GI of whole milk varies from 30 to 46 (out of 100), while low-fat or skimmed milk has a GI ranging from 20 to 34 [32]. The commercially available non-sweetened types of milk have a GL ranging between 2 and 5, which classifies them as a low GL food [32]. It should be noted that although milk is considered a low GI and GL food, the rise in blood insulin concentration during the 2 h after consumption, called the insulinemic index, is substantially higher than expected based on the GI of milk [33,34], indicating the presence of an insulinotropic ingredient, which is attributed to amino acids and lipids rather than the carbohydrates in milk products [35]. Persistent hyperinsulinemia potentially results in decreased insulin sensitivity [17], but it is also possible that the hyperinsulinemic response to milk has a positive or even protective impact on blood glucose regulation, especially in those with T2D [36,37]. Overall, the GI of milk means it could be a favorable component of a diet designed to manage blood glucose. To further explain the beneficial effect of milk carbohydrates on glucose metabolism, we will discuss the characteristics of lactose, galactose, and oligosaccharides of milk and their effects on glucose homeostasis in human trials (Table 1) and animal studies (Table 2).

### 2.1. Lactose

Lactose is a disaccharide of galactose and glucose (β-d-galactopyranosyl-(1→4)-d-glucose). In humans, the capacity to digest milk lactose is provided by lactase-phlorizin hydrolase (LPH aka lactase), a type of β-galactosidase, which is bound to the mucosal membrane of the small intestine. It hydrolyses lactose into glucose and galactose, which are then absorbed and carried to the liver by the portal vein [118]. This enzyme is encoded by the lactase (*LCT*) gene, located on chromosome 2q21; the activity of this gene is highest in infants. However, after weaning, decreasing abundance of this enzyme causes a gradual reduction in LPH activity in most humans, leading to a state of lactase non-persistence. Consequently, most adults are not able to digest lactose and may experience abdominal discomfort upon its consumption. Those who can digest milk lactose throughout life are considered lactase-persistent [119]. Substantial evidence supports that lactase persistence is influenced by at least five single nucleotide polymorphisms (SNPs) in a regulatory region called MCM6 (minichromosome maintenance complex component 6) upstream of the *LCT* gene [120]. Lactase persistence is prevalent among individuals of northern and Western European descent, as well as in many African, Middle Eastern, and southern Asian pastoralist communities. However, it is seldom persistent in other regions worldwide [121,122].

In contrast to sucrose, lactose intake is not related to the risk of T2DM development in human observational studies [123,124]. Human clinical trials show that lactose or lactose-derived prebiotics (e.g., lactulose) generally have beneficial or neutral effects on IR-related outcomes [38,39,40,125,126]. Lactose supplementation decreases body weight (BW) and fat accumulation in both healthy rats [64,67] and those with diet-induced obesity (DIO) [65]. These changes coincide with reduced circulating glucose, insulin, and leptin, indicating that lactose regulates metabolic processes [64,66,67].

Evidence supports that impaired glucose metabolism is, to some extent, triggered by elevated circulating bacterial endotoxins due to changes in intestinal permeability [127]. The presence of *Bifidobacteria* in the gastrointestinal tract is correlated with reduced plasma and intestinal endotoxin levels [128], which can ultimately reduce the detrimental effect of high-fat feeding elicited by endotoxins on glucose metabolism [129]. Furthermore, *Bifidobacteria* abundance correlates with reduced diabetes symptoms, including improved glucose tolerance [128]. This concept has been discussed in previous reviews [130,131]. In addition to being hydrolyzed and absorbed, lactose acts as a prebiotic [132], stimulating the growth and activity of beneficial bacteria in the gut and contributing to improved gut health [133,134,135]. As indicated earlier, many adults have low expression of *LCT* and difficulty digesting lactose, but even in lactase-persistent individuals, 5–10% of ingested lactose may escape digestion and pass through the lower intestine, producing digestion symptoms such as flatulence and diarrhea, depending on the degree of intolerance [136,137]. However, frequent lactose consumption promotes the growth of lactose-digesting bacteria, such as *Bifidobacteria*, as shown in a swine model [138]. These bacteria possess β-galactosidases that hydrolyze lactose into glucose and galactose, which are further converted into short-chain fatty acids, which have beneficial metabolic effects in obesity and other metabolic conditions [139,140,141]. Notably, the enrichment of *Bifidobacteria* has the added benefit of fermenting lactose without gas production, alleviating gut dysbiosis thus reducing intolerance symptoms [142].

Lactose significantly elevates calcium and magnesium absorption from milk and other foods because its fermentation lowers the pH in the large intestine, which increases the solubility and passive absorption of these minerals [35]. Calcium and magnesium play a role in the regulation of body weight and fat mass by reducing the de novo production of fatty acids, facilitating hydrolysis of stored fats, and creating insoluble complexes with dietary fats in the gastrointestinal tract, which reduce fat absorption [43], all leading to improved glucose homeostasis [35,44,45,46].

### 2.2. Galactose

Galactose, once hydrolyzed from lactose, is rapidly absorbed as a monosaccharide. The total galactose content per 100 g of milk, considering both the free galactose and that derived from lactose, ranges from 2.37–2.52 g [143]. Data on the average galactose intake in human populations are limited, with estimates ranging from 1.5–3.6 g per day in an Iranian female population [144] to below 0.5 g per day in a sample of Japanese women [145].

In human trials, galactose has drawn attention as a low-GI sugar because it causes very modest rises in plasma glucose and insulin concentrations in normal and T2DM participants [44,45], and also increases the secretion of incretins, glucagon-like peptide-1 (GLP-1) and glucose-independent insulinotropic peptide (GIP), which are produced in the small intestine [43,146], and promote satiety [46,147]. Both short- and long-term randomized controlled trials (RCT) in women who consumed galactose- rather than glucose-sweetened drinks report increased fat mobilization and oxidation [41], consistent with its smaller insulinemic effect [42,45]. 

Recent rodent studies shed light on the potential mechanism by which galactose improves glucose homeostasis. Providing galactose at 15% of dry matter for 9 weeks in non-diabetic, healthy rats increases hepatic insulin sensitivity and hepatic glycogen storage in the fed state, improves insulin capacity to decrease glycogen synthase phosphorylation, and reduces *Firmicutes* bacteria in the gut. Neither basal glucose nor insulin are modified by the dietary intervention [69]. Partial substitution of glucose with galactose in high-fat diet (HFD)-challenged female mice reduces BW, obesity, homeostatic model assessment of IR (HOMA-IR), and induces genes involved in insulin signaling in white adipose tissue depots, which could explain improved insulin sensitivity [68]. 

Galactose may also benefit liver carbohydrate metabolism. Nearly 90% of absorbed galactose is retained in the liver as galactose-1-phosphate (Gal-1-P), which serves as a substrate for glycogen production [148]. It can also be converted to alternative substrates (glucose, lactate, or fatty acids (FA)) that extrahepatic cells can easily utilize. Due to the delayed release of galactose into the peripheral circulation as glucose, it could be a preferred energy source compared with other monosaccharides because of its low GI and low insulinemic response [149]. 

Another mechanism by which galactose improves glucose homeostasis in rodents, as it does in humans, is by stimulating GLP-1 and GIP secretion into the circulation after a meal, which promotes insulin secretion to help maintain glucose homeostasis [70,146]. In addition, chronic galactose administration also restores a normal GIP and GLP-1 physiology in diabetes models [145,150]. Well-described IR- and T2DM-mitigating effects of GLP-1 are reported elsewhere [151,152]. The antioxidant and anti-inflammatory properties of galactose may also account for improvement in IR and obesity-related outcomes. Oral galactose supplementation reduces oxidative stress and inflammation markers in the liver of low-dose streptozotocin (STZ)-induced diabetes in rats [70]. 

### 2.3. Oligosaccharides

Cow’s milk oligosaccharides (CMOs) are complex carbohydrates consisting of 3 to 20 monosaccharides that are not digested but rather serve as prebiotics. Cow’s colostrum contains 1–2 g/L of CMOs; however, typical cow’s milk has significantly less CMOs (about 0.01–0.05 g/L). More than 100 different oligosaccharides are present in cow’s milk [153,154]. These include neutral oligosaccharides composed of glucose, galactose, and N-acetylglucosamine. Acidic oligosaccharides (sialyloligosaccharides) are attached to sialic and uronic acids. Lacto-N-biose, lacto-N-neotetraose (LNnT), lacto-N-triose (LNT), 3′-sialyllactose, 6′-sialyllactose, and disialyllactose are just a few of the oligosaccharides present in cow’s milk [21,155].

While human studies investigating CMO supplementation on glucose homeostasis are rare, animal studies provide promising results [71,72]. In DIO mice, CMOs improve glucose tolerance [72], reduce BW gain [71], decrease gut permeability [67,68] and systemic inflammation [72], and correct microbial dysbiosis [71,72]. Rats treated with HFD plus sialyloligosaccharides have improved insulin sensitivity and lower glycemic response in an oral glucose tolerance test (OGTT), along with higher adiponectin and lower leptin concentrations than the HFD controls [73]. Sialyloligosaccharides upregulate several genes involved in insulin signaling in both the liver and white adipose tissues, including *Gck*, *Kcnj11*, *Mtor*, *Pi3k*, and *Prckz*, while downregulating inflammatory markers such as *Iκbkβ* and *Mapk1* in the liver [73]. A mixture of CMOs improves the health and growth of undernourished mice pups and piglets by influencing their gut bacteria and metabolism, suggesting that CMOs, via the gut microbiota, have a significant impact on the development and regulation of metabolic pathways [75]. CMOs including tetrasaccharides LNT and LNnT promote the growth of short-chain fatty acid-generating bacteria [156] such as *Bifidobacterium*, *Lactobacillus*, and *Bacteroides* [157,158]. Supplementation with CMOs increases the expression of butyrate-generating bacterial genes in Western diet-fed mouse models, and butyrate has anti-inflammatory effects in the liver and colon [159]. 

## 3. Lipids 

The fat content of whole cow’s milk varies from about 3.0 to 6.0%, but is typically in the range of 3.5 to 4.7% and is comprised of more than 400 distinct FA, making milk the most complex and diverse dietary fat source. The composition of milk fat is influenced by the cow’s diet and the ruminal biohydrogenation process, as well as the breed, lactation stage, age of the cow, and the season and geographical location [160].

Milk fat consists of FA with carbon chain lengths ranging from 4 to 24. Palmitic acid (C16:0) and stearic acid (C18:0) make up the majority of saturated fatty acids (SFA) in milk fat, constituting around two-thirds of the total milk fat content [161]. Oleic acid (C18:1) is the predominant unsaturated FA. Milk fat also includes distinct FA produced by rumen microorganisms, such as trans isomers of octadecenoic acid ranging from 18:1 t4 to t16. Ruminant-derived milk and meat are the only sources of non-industrial trans-fatty acids (TFAs) in the human diet. Furthermore, the presence of branched-chain fatty acids (BCFAs) and odd-chain FAs, namely pentadecanoic acid (C15:0) and heptadecanoic acid (C17:0), in the unique FA composition of milk fat is noteworthy. Approximately 14% of the FA found in milk fat is classified as unique dairy-derived FAs and knowledge of their bioactivity is emerging [162]. 

The major lipids in cow’s milk include monoglycerides, diglycerides, triglycerides (TG), free fatty acids (FFA), phospholipids (PL), glycolipids, and sterols [162]. Milk fat is packaged inside a tri-layer milk fat globule membrane (MFGM), consisting of proteins, cholesterol, and polar lipids, which emulsifies and stabilizes the milk fat. Epidemiological evidence suggests that consuming dairy fat is neutral or beneficial in preventing chronic diseases [163]. This section focuses on bioactive lipids identified in cow’s milk and their effects on glucose metabolism (Table 1 and Table 2). 

### 3.1. Milk Fat Globule Membrane

MFGM contains both polar and neutral lipids and surrounds a TG core with an average diameter of 4  μm [164]. The polar lipids in MFGM included glycerophospholipids (e.g., phosphatidylcholine (PC)) and sphingolipids (e.g., sphingomyelin (SM)), which comprise about 1% of milk fat but about 25% of the components of MFGM. These polar lipids emulsify and stabilize TG within the aqueous phase of milk. Like other fats, milk TG undergoes hydrolysis by lipases to a mixture of FFA, mono- and di-glycerides in the intestinal lumen prior to its absorption by enterocytes, where it is repackaged into chylomicrons to be delivered to other metabolic tissues for subsequent uptake, storage or use [164,165,166,167]. At the same time, the PL of the MFGM, such as SM and PC, are also hydrolyzed by phospholipases. In addition, the polar lipids from the MFGM may become incorporated into enterocytes and influence gut physiology [168]. The industrialized processing of milk to remove fat and produce low-fat and fat-free dairy products leads to the loss of MFGM and a 40% decrease in total milk polar lipid. Both homogenization and pasteurization affect the rate of digestion, the size of MFGM, and its microstructure. Homogenized MFGM is digested more rapidly and releases more FA than native globules [169,170]. The intricate composition, segregation, and characteristics of MFGM have been thoroughly reviewed elsewhere [167].

Several RCTs show that MFGM positively impacts indicators of metabolic health and glucose homeostasis [47,48,49,171]. The inclusion of whipping cream enriched with intact MFGM in isoenergetic test meals high in saturated fat lowers the postprandial insulin response in a group of non-diabetic overweight and obese adults compared with the control meal [49]. Consistent with this finding, adding MFGM in palm oil reduces circulating insulin and glucose concentrations compared with palm oil alone [48]. In another RCT of isoenergetic diets, providing whipping cream (enriched with MFGM) for 8 weeks results in a slight increase in body mass index (BMI) among overweight adults compared with butter oil. However, there were no significant differences in fasting plasma glucose, insulin, and HOMA-IR between the groups [47]. In an RCT comparing MFGM-enriched milk beverage to a comparator beverage containing soy phospholipid and palm/coconut oil for 2 weeks, individuals with metabolic syndrome experienced no significant changes in fasting glucose, insulin, GLP-1, and HOMA-IR within or between the two treatment groups [50]. 

Multiple animal studies show consistent beneficial effects of MFGM feeding and illuminate potential mechanisms. Supplementation of MFGM for 8 weeks reduced glucose intolerance in a T2DM mouse model with the mechanism attributed to enhanced PI3K-AKT pathway and inhibited c-Jun N-terminal kinase (JNK) signaling in the insulin-resistant liver and skeletal muscle [80]. The JNK pathway plays an important role in insulin regulation because it inhibits AKT phosphorylation in skeletal muscle to accentuate IR [172,173]. Inhibition of JNK alleviates diabetes symptoms and improves insulin sensitivity in T2DM rats [174]. The further beneficial impact of MFGM on glucose regulation is elucidated in the early exposure of offspring to MFGM [79]. The administration of MFGM HFD-induced obese rats during pregnancy and lactation resulted in the amelioration of HFD-induced ectopic fat accumulation, in addition to an improvement in insulin resistance by inducing p-AKT and reducing p-IRS in adult offspring [78]. In another study, Ye et al. showed an improvement in glucose tolerance without significantly impacting the weight gain in C57BL/6 mice offspring exposed to maternal HFD when supplemented with MFGM [81]. They attributed this effect to the presence of beneficial bacteria in the gut. 

Regarding body composition and weight gain in the presence of IR, MFGM treatment elicits promising results in animal studies. Obese, pregnant female rats fed MFGM have reduced BW, enhanced glucose tolerance and insulin sensitivity, and restored expression of genes involved in insulin signaling in multiple tissues [77]. MFGM-supplemented animals have reduced BW gain and obesity [77], possibly explained by upregulated thermogenic genes such as *Ucp1* and *Cidea* to increase brown adipose activity and browning of white adipose tissue [76]. In cultured human HepG2 cells, MFGM exposure mitigates the accumulation of lipid droplets and maintains higher glucose uptake when compared to control cells, concomitant with an increased abundance of glucoregulatory enzymes such as glucose-6-phosphatase, glyceraldehyde-3-phosphate dehydrogenase, glycogen phosphorylase, and hexokinase, and consistent with the improvement in insulin signaling and glucose metabolism [175].

The presence of glycerophospholipids and sphingolipids in MFGM may mediate its effects on metabolism [14]. Subclasses of glycerophospholipids include PC, phosphatidylethanolamine, phosphatidylserine, and phosphatidylinositol. The sphingolipids include SM and glycosphingolipids. SM, PC, and phosphatidylethanolamine are the most abundant polar lipids in the MFGM, constituting approximately 30%, 30%, and 20% of milk polar lipids, respectively. Phosphatidylserine and phosphatidylinositol are less abundant, each making up less than 10% of milk polar lipids [176].

In a human trial, an RCT of 62 overweight or obese men demonstrated the impact of daily intake of 2 g of PL-enriched milk to elicit reduced waist circumference compared to unenriched milk fat for 8 weeks. However, 57 male participants receiving milk 3 g of PL-enriched milk for 7 weeks showed no significant differences in fasting glucose, insulin, or the insulin sensitivity index compared to those receiving 2.8 g soy PL [51].

Several research studies indicate that milk polar lipids improve metabolic diseases through regulation of the gut microbiota [52,77,83,84,88,176]. Adding 1.6% milk PL (0.38% SM) to an HFD resulted in a decrease in fecal *Lactobacillus* in C57BL/6 mice, whereas 1.1% milk PL (0.25% SM) increased *Bifidobacterium* compared to the control HFD group [82]. *Akkermansia muciniphila* increased in mice fed with milk PL, enhancing insulin sensitivity and protecting against inflammation caused by metabolic endotoxemia [177,178]. The source of SM may determine its ability to influence metabolism, as two studies using milk SM found no effects on BW, adiposity [87], blood glucose [86,87] or HOMA-IR [86] in mouse models. However, egg-derived SM did lower fasting glucose levels [86]. 

As mentioned previously, conditions such as obesity and IR are interconnected with gut health and the ability of the intestinal barrier to prevent the translocation of endotoxins that trigger inflammation; these actions are mediated via the Toll-like receptor (TLR)-4/nuclear factor (NF)-κB pathway [129,179,180]. In neonatal rodents, milk polar lipids derived from buttermilk enhance gut barrier function by reducing intestinal inflammation typically induced by the TLR-4-NF-κB pathway [181]. Additionally, milk polar lipids protect mice against impairments in gut barrier integrity caused by lipopolysaccharides [182]. In cell-based assays, PL- or ganglioside-containing fractions of the MFGM reduce inflammatory responses such as neutrophil elastase activity, superoxide production, and IL-1β release [183]. These findings highlight the potential role of milk-derived polar lipids in modulating gut health and inflammation, which often has implications for glucose homeostasis.

Nagasawa et al. identified interesting effects of dihydrosphingosine, a product of SM hydrolysis, on the activation of GPR120, a receptor expressed by enteroendocrine cells that facilitates the release of the incretin GLP-1 [184]. In vitro, dihydrosphingosine in conjunction with phytosphingosine exhibited potent activation of GPR120, although sphingosine did not [185]. Further research is necessary to explore the potential of milk polar lipids in modulating incretin production.

### 3.2. Fatty Acids

Milk is a source of FAs of varied length and saturation, which individually may have contrasting effects on metabolic health. FA species are classified as saturated and unsaturated odd-chain (OCFA), even-chain (ECFA), monounsaturated, polyunsaturated, branched-chain (BCFA), and TFA [186]. Except for the trans species, which include conjugated linoleic acid (CLA), rumenic acid (RA) and trans-11 vaccenic acid (VA), these FAs are not uniquely present in cow’s milk. This section will briefly summarize the general effects of cow’s milk FAs on glucose homeostasis, focusing on OCFA and TFA with a brief summary of the other classes.

#### 3.2.1. Even-Chain Fatty Acids

The major saturated ECFA in whole milk includes myristic, palmitic, and stearic acid, respectively C14:0 (0.3 g/100 g), C16:0 (0.8 g/100 g), and C18:0 (0.4 g/100 g), with C10:0 and C12:0 contributing less than 0.2 g/100 g. ECFA makes up approximately 68% of FA present in cow’s milk [90,186]. 

Numerous studies report associations between consumption of ECFAs C14:0, C16:0, and C18:0 (not specific to milk) and increased risk of T2DM, obesity, IR, and metabolic dysfunction [89,187,188]. C16:0 and C14:0 feeding elicits increases in BW, reduced insulin secretion, hepatic steatosis, and increased endoplasmic reticulum stress in mouse adipose tissue and insulin-secreting INS-1E cells [89,189]. Clarifying whether ECFAs from dairy products confer different effects because of the unique dairy matrix compared to other dietary sources could help to allay concerns regarding whole milk [5,6]. Indeed, recent comprehensive systematic reviews [4] and large-scale observational studies [190,191] find, at minimum, no harm and potentially a benefit of consuming dairy fat on cardiovascular risk factors.

#### 3.2.2. Odd-Chain Fatty Acids

The saturated OCFAs are pentadecanoic acid (C15:0) and heptadecanoic acid (C17:0), which comprise only 1.5% of FA in milk [90]. They are produced by rumen microbial fermentation and microbial de novo lipogenesis. The synthesis of these FAs by human enzymes is minute, rendering them dependable biomarkers of dairy fat consumption [192]. C15:0 and C17:0 FA concentrations in milk are approximately 0.9 g/100 g and 0.5 g/100 g, respectively. Their concentration in the blood is proposed as an indicator of the fat content of the consumed dairy products to distinguish between the consumption of low-fat or fat-free dairy products versus whole-milk dairy products [193]. 

The negative association of higher circulating concentrations of OCFAs with lower risk of cardiometabolic disorders indicates potential health advantages that extend beyond their application as biomarkers [194]. Observational data in humans demonstrates a lower risk of T2DM associated with higher proportions of C15:0 and C17:0 in erythrocyte membranes [195]. Furthermore, studies report a negative correlation between metabolic disease risk and circulating C15:0 and C17:0 [91,196]. Although the inverse associations of C15:0 with metabolic disease risk are consistently supported [197,198,199], the evidence for C17:0 is more variable [197]. Experiments to determine if the benefits of OCFA counterbalance ECFA effects when consumed within the dairy matrix would help clarify why dairy products do not appear to have the same detrimental effects as other foods rich in long-chain ECFA. 

A recent 12-week RCT of 88 women with metabolic dysfunction-associated steatotic liver disease (MASLD) compares the effects of a modified Mediterranean diet with and without 300 mg C15:0 supplementation to a control group maintaining a habitual diet. BMI, liver fat content, and various metabolic risk factors are improved in both diet intervention groups compared to the control group and are more pronounced in the diet with C15:0 supplementation [53]. The primary outcomes do not directly measure specific effects of C15:0 supplementation on glucose homeostasis. However, given the overall metabolic improvements and potential gut microbiome alterations induced by C15:0 supplementation, such as increased abundance of *B. adolescentis* [53], it is plausible that these changes could indirectly influence glucose regulation.

In preclinical investigations utilizing diverse human cell lines and rodent models, consistent beneficial outcomes on metabolic parameters are observed, particularly for C15:0. In cultured mouse myotubes, C15:0 treatment increases glucose uptake and glucose transporter 4 (GLUT4) translocation to the cell membrane, especially when administered with insulin, suggesting a significant enhancement of insulin sensitivity [200]. GLUT4 is the major glucose transporter responsible for glucose uptake in muscle and adipose cells, and its translocation to the cell surface is associated with increased glucose uptake from the circulation, reflected in normalized blood glucose levels and, probably, improved insulin sensitivity [201]. The mechanism behind increased GLUT4 translocation involves the activation of 5′ AMP-activated protein kinase (AMPK) and AKT substrate 160 (AS160) [200]. In human cell culture studies, 10 to 50 μM of C15:0 is a partial agonist of peroxisome proliferator-activated receptors (PPAR) α/δ. It also lowers mitochondrial reactive oxygen species production in HepG2 cells [90], underscoring its capacity to enhance mitochondrial function. This is vital for maintaining hepatocyte function and metabolic homeostasis, including glucose regulation. Furthermore, C15:0 activates anti-inflammatory and antifibrotic pathways in cell cultures representing multiple tissues, as shown by reduced pro-inflammatory markers such as monocyte chemoattractant protein 1 MCP-1 and secreted immunoglobulin G, alongside decreased fibrosis indicators like collagen I and plasminogen activator inhibitor 1. In an HFD rat model, C15:0 supplementation reduces proinflammatory cytokines and lowers fasting blood glucose and BW gain [90]. These results highlight its potential in mitigating chronic inflammation and fibrosis, conditions often associated with metabolic disorders including IR and impaired glucose homeostasis [90].

Overall, dairy-derived C15:0 mediates improved glucoregulation by reducing dyslipidemia, promoting mitochondrial repair, and decreasing inflammation. Indeed, some researchers argue that C15:0 could be an essential FA [90,202]. In contrast, in HFD-fed male rats, C17:0 elicits no phenotypic changes [90]. Likewise, in HFD-fed male mice supplemented with either dairy fat or C17:0, exacerbated liver inflammation is detected with no improvement in insulin sensitivity [92]. However, for both OCFAs, more in vivo data are required.

#### 3.2.3. Trans Fatty Acids

TFAs naturally occurring in milk constitute 4–6% of the overall fat content (~0.1 g per 100 mL of whole milk). VA (18:1, n-7) is the most abundant TFA in dairy milk fat, with relative amounts of total TFA varying depending on the cows’ diet [161,203].

Several in vivo and in vitro studies demonstrate the beneficial impacts of VA on metabolic endpoints. In T2DM male Sprague Dawley rats, 1.0% *w*/*w* VA supplementation in a diet containing butter oil as the dairy background improves fasting blood glucose and glucose-stimulated insulin secretion in vivo and from isolated islets [94], which is associated with increased β-cell area and up-regulation of the FA receptor GPR40 in islets [94]. VA supplementation also elicits greater total energy expenditure accompanied by an enhanced respiratory exchange ratio, indicating improvement in substrate utilization in a rat model of MASLD and metabolic syndrome [93]. Supplementing 1.0% VA in the diet reduces the mRNA of *Fas* and *Acaca* in the liver, which may contribute to the observed decrease in MASLD symptoms and also reduce the amount of visceral adipose tissue in obese JCR:LA-cp rats [93]. Similar results occur in fa/fa Zucker rats fed with 1.5% (*w*/*w*) VA [96]. The observed impact on visceral adipose tissue may be via VA acting as a ligand for both PPAR-α and PPAR-γ [204]. Despite these encouraging findings, in human studies, providing diets enriched in VA for 4–5 weeks had no significant effect on insulinemia and glycemia in either healthy men or overweight women [205,206].

CLA has garnered significant scientific and general public attention because of its putative modulation of cardiometabolic risk factors, including obesity, inflammatory indicators, IR, glucose metabolism, and diabetes [207]. The primary source of CLA in cow’s milk is biohydrogenation of linoleic acid by microorganisms in the rumen [208]. Pasture-based diets enhance the concentration of CLA in milk because of the linoleic acid precursor present in the forage [208,209]. In addition, a substantial proportion of CLA present in milk is produced in the mammary gland of the cow from VA [209].

Despite the attention, results in human studies are mixed. A meta-analysis of 13 RCTs reveals a notable rise in fasting blood glucose after administering CLA to human participants with a high cardiovascular disease risk, but the results are highly heterogeneous. In addition, no significant impact is seen on hemoglobin A1c (HbA1c) or HOMA-IR compared to the control groups [56]. Other meta-analyses examining the effects of CLA on IR and risk factors associated with diabetes show its potential to decrease key metabolic health indicators, such as inflammatory markers interleukin-6 and tumor necrosis factor-α [210], BMI and body composition [54,55], and oxidative stress markers [211]. Thus, CLA supplementation, as part of a strategy to mitigate risk factors linked to IR and diabetes, may be beneficial [212] but effects on overall cardiovascular risk are less impressive. One reason for the divergent results may be because CLA consists of a mixture of isomers.

Limited clinical studies of the isomer RA on human health outcomes, particularly in relation to glucose homeostasis, demonstrate mixed results. A cross-over design RCT of healthy individuals, providing meals substituting 3% VA, 3% industrial CLA isomers, or 1% RA for stearic acid each for 24 days finds no significant differences among the dietary treatments on blood glucose, insulin, or HOMA-IR [57]. In contrast, RA supplementation in males with obesity (approximately 1% of energy intake for 12 weeks) reduces insulin sensitivity compared to placebo [213], thus raising concerns about the impact of RA on metabolic health. 

Most of the evidence for the health benefits of CLA or RA, including their hypothesized antidiabetic capabilities, is from studies in animal and in vitro models. In rodents, CLA supplementation has inconsistent IR-related outcomes, yielding worsened insulin sensitivity in a lactating mouse study [98] but improving OGTT in a HFD-rat model [99]. In the latter study, recovery of *Ucp2* and *Ucp3* mRNA expression in muscle suggests increased energy expenditure as a mechanism. RA-enriched butter significantly increases fasting serum insulin in male Wistar rats fed an HFD [101]. In female C57Bl/6J mice, 6 months of supplementation with RA (0.5% of total fat) increases lean mass and decreases fat mass, and notably decreases glucose, HOMA-IR, and improves insulin sensitivity, consistent with reduced IR [97]. Similarly, ob/ob mice provided RA have reduced plasma insulin, glucose and HOMA-IR and an improvement in insulin sensitivity [100]. 

RA mimics the ability of insulin to upregulate GLUT4 trafficking to the plasma membrane and increase glucose uptake in L6 myotubes upon acute exposure to RA [214]. Also, the exposure of HepG2 liver cells to RA inhibits gluconeogenesis by reducing key enzymes *Pck1* and *G6pc1* expression [215].

#### 3.2.4. Branched-Chain Fatty Acids

BCFAs in milk are typically SFAs with one or multiple methyl branches along their carbon chains. The structural forms of BCFA are characterized by a branch point on the second-to-last carbon atom in iso BCFAs and a branch on the third-to-last carbon in ante-iso BCFAs. Microorganisms in the rumen utilize the dietary branched-chain amino acids (BCAA) valine, leucine, and isoleucine to produce BCFA. BCFAs contribute 1.7–3.4% of the total fatty acids in milk and consuming three servings of whole milk per day provides 367–763 mg of BCFA [216]. Monomethyl BCFAs with chain lengths of 14–17 carbons predominate, featuring either iso or ante-iso configurations. The most abundant milk BCFA is ante-iso C15:0, followed by iso C17:0, iso C15:0, ante-iso C17:0, and iso C16:0 [217]. Multimethyl BCFAs, specifically phytanic acid (0.1–0.5%, or 7–37 mg per serving of whole milk) and its derivative pristanic acid (0.04–0.06% or 3–4 mg per serving of whole milk), are minor components of milk fat [218,219]. The origin and metabolism of BCFAs has been reviewed and elaborated elsewhere [216].

Recent observational studies highlight a significant association between BCFA intake and energy and glucose homeostasis. Total BCFAs in serum are higher in non-obese compared to obese women, with iso-BCFAs displaying an inverse association with BMI [220]. Circulating BCFAs are associated with higher weight loss and possibly less body fat accumulation after gastric bypass surgery in people with obesity [221]. Furthermore, the proportions of total and individual BCFAs in the adipose tissue of lean subjects are higher than those of obese individuals [222], associated with reduced prevalence of dysglycemia [223]. 

The liver content of BCFA of female C57BL/6J mice is inversely correlated with total hepatic fat accumulation, suggesting that BCFAs may play a role in lipid metabolism regulation [224]. A recent in vitro study using a human fatty liver cell line (L02 cells) supports this observation, showing that iso-15:0 and iso-18:0 BCFAs reduce cellular TG and upregulate genes involved in lipid catabolism [225]. In the rat insulinoma INS-1 β-cell line (a well-established model for studies of pancreatic islet β-cell function) BCFA iso-17:0 upregulates critical transcription factors for optimal insulin secretion like PDX1 and PPAR-γ [102]. 

Dietary BCFAs are hypothesized to decrease inflammation, which is an underlying cause of glucose dysregulation. In humans, an inverse association is observed between serum iso-BCFAs (e.g., iso-15:0, iso-16:0, iso-17:0, and anteiso-15:0) and C-reactive protein, an important inflammatory marker associated with the risk of T2DM [220]. Sprague Dawley rat pups that consume a diet containing a mix of BCFAs have enhanced expression of interleukin-10, an anti-inflammatory cytokine [226]. Also, BCFA reduces the mRNA of key pro-inflammatory mediators such as *Cxcl8*, *Tlr4*, and *Nfkb1* in a model of lipopolysaccharide-induced inflammation in gastrointestinal Caco-2 cells [227]. While most studies to date intervened with a combination of BCFAs, thoroughly investigating milk-derived BCFAs and specific milk BCFA species, such as iso-15:0 and iso-17:0, is needed in both in vivo and in vitro models to elucidate their effects on glucose homeostasis to help in developing targeted dietary recommendations and therapeutic interventions involving BCFA.

## 4. Protein Hydrolysates and Peptides

Milk proteins are nutritionally rich because they have an adequate balance of essential amino acids. Cow’s milk has a total protein content of about 3.5% by weight yielding 21% of the calories in whole milk. Caseins (specifically α-, β-, γ- and κ-casein) and wheys (several globulins, including α- and β-lactoglobulin) are the primary classes of protein found in milk, in a ratio of 4:1. Membrane globular proteins, glycoproteins, and lipoproteins mainly constitute the outer layer of MFGM. These proteins constitute less than 2% of the overall protein content [228,229]. 

Ingestion of intact whey has acute and chronic effects on glycemia. Indeed, a meta-analysis showed that whey consumption reduces the area under the curve of a glucose tolerance test concurrent with enhanced insulinemic response in both healthy people and those with T2D [19]. Chronically, whey reduced fasting insulin, FBG, and HOMA-IR in a sub-analysis pooling the results of 36 RCTs [230], consistent with three other recent meta-analyses [231,232,233,234,235]. The sub-analysis of Mohammadi suggested casein was less effective than whey in modulating glycemic parameters [230].

Milk is an excellent source of bioactive peptides. Bioactive peptides are usually 2–20 amino acids in length and, besides their nutritional value, exert beneficial physiological effects [236]. Bioactive peptides derived from milk are initially inactive within the primary structure of milk protein. The bioactive forms are primarily generated through proteolysis of casein and whey proteins [237,238,239], but β-lactoglobulin hydrolysis also generates bioactive peptides, such as wheylin [240,241].

The most well-researched milk-derived bioactive peptides are valine-proline-proline (VPP) and isoleucine-proline-proline (IPP) [242]. Although mostly tested for their anti-hypertensive effects [243,244,245,246], milk-derived hydrolysates can improve IR and modulate glucose tolerance in humans [63,247,248]. However, a meta-analysis of human trials found no consistent effect of casein hydrolysate on cardiovascular risk factors, showing that despite significantly lowering blood pressure, there was no significant effect of FBG [249]. Another meta-analysis included the combination of protein hydrolysates from different sources, indicating a significant reduction in postprandial blood glucose response among adults with normal glycemia as well as those with hyperglycemia [250]. Herein, we review clinical and preclinical studies that utilized milk hydrolysates and peptides and their effect on IR and glucose-related measurements but do not consider total protein from milk, such as whey isolates, casein isolates, or whey proteins.

In general, dietary supplementation with milk-derived bioactive peptides/hydrolysates does not affect food intake in animals. However, one study shows that acute, but not chronic, diet supplementation with a casein-derived peptide (CHM-273S–sequence: SKDIGSESTEDQAME) reduces food intake in C57BL/6 mice [113] and others find that whey hydrolysate decreases food intake compared to a chow diet in rats [104]. 

### 4.1. Whey Hydrolysate

Whey hydrolysates comprised of smaller peptides and free amino acids are prepared by enzyme digestion at acidic or alkaline pH [251,252]. In a randomized, cross-over trial in patients with prediabetes, acute provision of 1400 mg of whey hydrolysate before a carbohydrate-rich challenge meal significantly decreased the postprandial elevation of blood glucose as compared to the placebo, indicating better management of blood glucose. Long-term usage markedly decreased HbA1c but did not further improve OGTT. Twice the dose did not improve any outcome [63]. In an RCT conducted on overweight/obese women, the group that received a combination of an energy-restricted diet and whey hydrolysate (20 g) for 12 weeks had reduced BMI and body fat when compared to energy restriction alone [59]. In contrast, a 12-week intervention with 60 g/day of whey hydrolysate in adults with abdominal obesity did not result in changes to the HOMA-IR [62].

Among the animal studies using whey hydrolysate and investigating insulin sensitivity, signaling, or glucose tolerance [103,104,105,106,107,108,110], consistent improvements in glucose tolerance are demonstrated; however, the mechanisms identified are different in each study, depending on the pathways investigated. Zucker diabetic fatty (ZDF) rats fed whey hydrolysate (13 weeks) on a background of chow diet exhibit improved OGTT and lowered HbA1c versus controls. The hydrolysate has no effect on these parameters in lean Wistar rats or on fasting glucose or insulin in either ZDF or Wistar rats [104]. The authors hypothesize that the effects observed in ZDF rats could be due to lower circulating glucagon, which would lead to decreased hepatic gluconeogenesis [104]. In ob/ob mice, whey hydrolysate supplementation for 8 weeks improves OGTT and decreases fasting insulin and HOMA-IR. These effects are attributed to higher insulin-secreting capacity, as measured from isolated pancreatic islets [106], perhaps mediated by increased plasma concentration of insulinotropic amino acids following enhanced intestinal absorption. Lean, insulin-sensitive mice exhibited improved OGTT but no other changes [106]. A short-term treatment with whey hydrolysate and exercise intervention in Wistar rats elicits no changes in fasting insulin compared to controls, but the hydrolysate-treated animals have increased liver and skeletal muscle glycogen content. In addition, up-regulated skeletal muscle GLUT4 translocation to the plasma membrane and p-AKT (indicating insulin signaling) is more pronounced in whey + exercise rats [103]. The combined results of these studies indicate that whey hydrolysates affect both insulin secretion and signaling to improve glucose homeostasis. However, a minority of studies do not find benefit. For example, in C57BL/6 mice, whey hydrolysate feeding for 13 weeks exacerbates the effects of HFD, including increased BW, impaired glucose tolerance, and increased HOMA-IR, while having a null effect on insulin sensitivity [105]. These effects are accompanied by ectopic lipid accumulation and lower mitochondrial oxidative phosphorylation in skeletal muscle, and PPAR−α suppression in WAT, which can contribute to IR [105].

Whey hydrolysate may influence glucose homeostasis by modulating intestinal hormones. In vitro studies simulating gastrointestinal digestion demonstrate that whey hydrolysate enhances the synthesis and processing of GLP-1 more than intact whey isolate. Furthermore, whey hydrolysate reduces dipeptidyl peptidase-IV (DPP-IV) activity in a cell model [253]. GLP-1, an incretin hormone involved in glucose control, has a relatively short half-life due to the activity of DPP-IV, which is present in both cell-associated and circulatory states and is highly expressed in blood and enterocytes. The involvement of DPP-IV in maintaining glucose homeostasis entails the deactivation of incretins, GIP, and GLP-1 [247]. 

### 4.2. Casein Hydrolysate

In adults with T2DM, casein hydrolysate interventions consistently improve glucoregulation by increasing insulin secretion. Compared with placebo, consumption of casein hydrolysate (0.3 g/kg) in conjunction with leucine after a main meal by people with T2DM results in a substantial decrease in the mean 24-h blood glucose and increased circulating insulin [60]. Another study demonstrates enhanced de novo insulin production in participants with T2DM supplemented with casein hydrolysate (0.35 g/kg) in comparison to the consumption of a control carbohydrate diet [254], effects that are maintained even in the absence of supplementary amino acids [248]. In another RCT, including patients with T2DM, an OGTT was conducted after acute administration of casein hydrolysate at a dose of 0, 6, or 12 g. The highest dose resulted in elevated insulin and decreased glucose levels post-challenge [61]. 

Casein hydrolysate supplementation also demonstrates beneficial effects on insulin signaling and glucose handling in rodents. Long-term casein hydrolysate supplementation in an HFD-induced obese mouse model improved glucose tolerance [110]. Although no changes are seen in an insulin tolerance test (ITT), insulin signaling is enhanced in skeletal muscle, liver, and WAT via increased AKT activation. Less pronounced or null changes in vivo are seen in chow diet-supplemented animals [110]. Casein hydrolysates (4%) administered to mice with HFD-induced obesity decreased systemic inflammation induced by the HFD and improved ITT (i.e., IR), reduced fasting insulin and HOMA-IR, although FBG was unchanged compared with the HFD group [111]. Conversely, in a T2DM mouse model, 8 weeks of casein hydrolysate recovered BW lost after STZ treatment without affecting food intake [107]. FBG and OGTT were decreased, but no changes in fasting insulin were observed. The casein hydrolysate increases skeletal muscle glycogen content and enhances muscle synthesis protein activation via phosphorylation of glycogen synthase kinase-3β. Moreover, insulin signaling is enhanced with increased phosphorylation of IRS-1, PI3K, and AKT and GLUT4 translocation to the plasma membrane [107]. Of note, the authors report casein hydrolysate modifies the gut microbiome, which may contribute to the benefits. In DIO C57Bl/6 mice, casein hydrolysate decreased BW, FBG, insulin, glucagon, and leptin, with no changes in the OGTT after 7–8 weeks of supplementation [108]. This is accompanied by decreased adipocyte size, higher ex vivo FA oxidation in subcutaneous WAT, and increased expression of browning markers such as *Ucp1*, *Cox8b*, and *Mpzl-2* in this model, which might increase energy expenditure [108]. Finally, β-lactoglobulin hydrolysate provided acutely to KK-A^y^ mice yielded improvements in both OGTT and ITT accompanied by increased p-AKT in the liver and skeletal muscle [109], but more studies of this hydrolysate are required. 

The bioactivity of protein hydrolysates depends on the combinations of enzymes used. Oral administration of casein hydrolysate produced using a combination of papain and Flavourzyme resulted in a drop in FBG and HbA1c, and improvement in OGTT, in rats with T2DM caused by STZ and an HFD compared with hydrolysates prepared with papain or Flavourzyme alone. All treatment groups experienced an increase in circulating insulin and HOMA-β (a measure of β-cell function) compared with the control group [255]. Furthermore, the administration of Flavourzyme-papain hydrolysate increases phosphorylation of AMPK and the expression of *Glut2*, along with inhibition of phosphoenolpyruvate carboxylase kinase activity and an elevation in glycogen content in the liver [255]. Others report that Flavourzyme-papain casein hydrolysates reduce liver oxidative damage by increasing the transcription of *Nrf2*, which upregulates antioxidant activity. It was proposed that such treatment could be a viable option for managing liver damage in individuals with T2DM [112].

In cell culture experiments, casein hydrolysates dose-dependently inhibit mitogen-activated protein kinase (MAPK)-c-JNK phosphorylation and increase the phosphorylation of ERK in TNF-α-induced insulin-resistant 3T3-L1 adipocytes, consistent with the potential to improve chronic inflammation in WAT [111]. In HepG2 liver cells, glucose uptake was enhanced by Flavourzyme-papain casein hydrolysates in the absence of insulin, an activity attenuated by the application of inhibitors targeting both the AKT and AMPK pathways, whereas the AMPK signaling route was exclusively activated by Flavourzyme-papain hydrolysates in T2DM rats (see above) [255]. C2C12 myotubes, a model of skeletal muscle incubated with casein hydrolysate, have enhanced 2-deoxy-glucose uptake compared with untreated cells, explained by increased p-AMPK and liver kinase B1 [256]. 

Overall, casein hydrolysate consistently improves endpoints related to glucose homeostasis in both human and animal studies. The mechanisms may include enhanced insulin secretion and insulin signaling in the liver, adipose, and skeletal muscle. Hydrolysates prepared with papain and Flavourzyme in combination show efficacy in activating cell pathways that regulate glucose uptake in multiple trials. 

### 4.3. Bioactive Peptides

The significance of milk-derived peptides in human physiology and health remains relatively uncertain because our understanding of them is mostly based on preclinical studies. Studies investigating specific peptides derived from milk in rodents [109,113,114,115,116,117] exhibit consistent improvement in glucose tolerance, insulin signaling, and insulin sensitivity. For example, dietary supplementation with a glycomacropeptide derived from casein for 12 weeks decreased insulin and HOMA-IR but did not protect against high-fat/high-sugar diet-induced obesity or improve OGTT in C57BL/6 mice [115]. This casein-derived glycomacropeptide increases liver insulin signaling through phospho-AKT, modulates the abundance of proteins involved in lipid metabolism and gluconeogenesis via increased phospho-acetyl CoA carboxylase, PPAR-α, PPAR-γ coactivator-1α and carnitine palmitoyl transferase-1α, and decreases fatty acid synthase, phosphoenolpyruvate carboxykinase and glucose-6-phosphatase [115]. A different casein-derived peptide (CHM-273S–sequence: SKDIGSESTEDQAME) acutely elicited improved glucose tolerance in Sprague Dawley rats associated with enhanced liver insulin signaling through the AKT pathway [113]. In C57BL/6 mice, the same peptide provided chronically in the diet reduced BW, visceral fat mass, adipocyte size, fasting glucose, and HOMA-IR despite no changes in fasting insulin. Its acute administration to mice does not elicit any improvements in glucose tolerance, which suggests its effects are mediated by metabolic adaptations [113].

VPP, a well-studied milk-derived peptide with anti-hypertensive properties, showed no effects on BW, fasting glucose, or fasting insulin but improved ITT and white adipose tissue inflammation after 16 weeks of supplementation in HFD-fed C57BL/6 mice. These effects are attributed to the renin-angiotensin system inhibitory activity of the peptide [116]. Another casein-derived peptide supplemented prior to exercise training led to decreased BW gain, fat mass, FBG and HOMA-IR after 4 weeks in HFD mice. OGTT was improved in treated animals, along with increased GLUT4 protein content in skeletal muscle [114]. A bioactive dipeptide derived from β-lactoglobulin called wheylin-1 (sequence: MH) improved ITT and enhances p-AKT in the liver of KK-Ay mice [109]. In normal mice, acute administration of a casein-derived peptide (VPYPQ) improved OGTT, an effect attributed to DPP-IV inhibitory activity seen in vitro [117].

Individual amino acids found in milk proteins may also influence glycemic parameters. Compiled evidence indicates that higher circulating branched-chain amino acids (BCAAs), which include leucine, isoleucine, and valine, may increase the risk of T2D [257], whereas reduced circulating BCAAs demonstrate alleviation of insulin resistance [258]. BCAAs may influence both insulin sensitivity and insulin secretion [259,260,261]. However, the impact of dietary intake of BCAAs is an ongoing debate that has been comprehensively evaluated in current reviews [262,263,264]. Their effects in isolation may be distinct from the ingestion of intact proteins, hydrolysates, or peptides. Indeed, the n-terminal amino acid of both VPP and IPP is a BCAA. Another example is isoleucine-arginine-tryptophan, an egg white-derived tripeptide, which improves glucose tolerance and insulin sensitivity in rats and mice despite one of its constituents being a BCAA [265,266].

Overall, milk-derived hydrolysates and peptides induce relatively consistent improvements in IR, insulin signaling, and glucose tolerance in rodents. Variability in response to hydrolysates likely stems from the variety of tested doses, ranging from 1 mg/mL of water [105] or 100–200 mg/Kg BW [106,107] in the case of casein hydrolysates. Also, differences in species/strains of rodents and background diet (HFD or low-fat/chow diet) confound results. Studies utilizing specific peptides have demonstrated more consistent results, particularly in the enhancement of insulin signaling at the tissue level. However, single peptides in vivo could be less effective than hydrolysates if the peptides within the mixture provide synergistic effects. A summary of the actions of the peptides and hydrolysates leading to improvements observed in the studies outlined is illustrated in Table 2.

## 5. Summary and Conclusions

**Figure 1:**
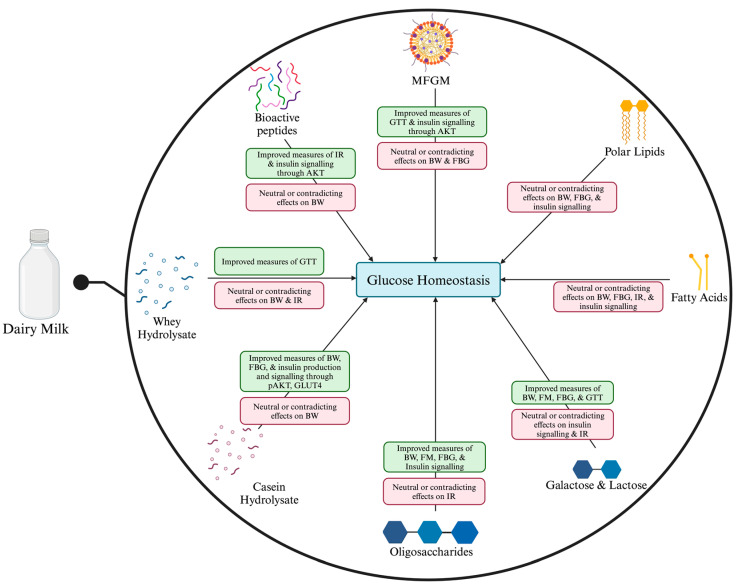
A summary of the evidence that bioactive compounds from milk can regulate glucose homeostasis. Abbreviations: BW, body weight; GTT, glucose tolerance test; IR, insulin resistance; AKT, protein kinase B; FBG, fasting blood (plasma) glucose; FM, fat mass; GLUT4, glucose transporter-4. Created with BioRender.com (accessed on 30 August 2024).

This review highlights the multifaceted role of cow’s milk components in modulating glucose homeostasis and insulin sensitivity, which could be utilized to prevent and manage T2DM. The primary carbohydrate in milk, lactose, has a low GI and does not raise blood glucose compared to other simple sugars, which is beneficial for maintaining stable blood glucose. Furthermore, the prebiotic properties of lactose and oligosaccharides promote the growth of beneficial gut bacteria, contributing to improved gut health and potentially enhancing glucose metabolism through mechanisms that reduce inflammation. The FA profile of milk includes a complex array of saturated and unsaturated fats that have diverse effects on health. Notably, the MFGM and certain FAs, such as odd-chain C15:0 and VA, are associated with positive metabolic outcomes. These components may improve insulin sensitivity and modulate lipid metabolism and inflammation, but animal results are not consistently replicated in human trials. The bioactive peptides derived from casein and whey influence insulin secretion and sensitivity, which are essential mechanisms for maintaining normal blood glucose. They may also enhance incretin activity by inhibiting DPP-IV. An overall summary is provided in Figure 1.

Although cow’s milk is the most popular milk consumed by the majority of people, the health impacts of milk from other ruminant species, including goats, sheep, and camels, has received much attention because their consumption in a particular region or country may be high [267,268]. Similar to cow’s milk, other ruminants’ milk is also rich in various bioactive molecules that may overlap with cow’s milk or be specific to the species [269,270]. For example, bioactive molecules in sheep’s milk, including CLA and lactoferrin, can enhance insulin sensitivity and reduce inflammation, similar to the effects observed with cow’s milk [271]. Given the particular composition of its medium-chain fatty acids, goat’s milk holds promise for improving lipid metabolism and reducing hyperglycemia [272]. Furthermore, camel’s milk, rich in insulin-like proteins, was shown to decrease glycemia while improving insulin secretion, thus possibly having therapeutic benefits in the management of T2DM [273]. These comparative insights underline the potential of the bioactive molecules in the milk of different species in therapeutic interventions, specifically in modulating glucose homeostasis [15,274,275,276,277]. 

In conclusion, it is evident that milk-derived bioactive molecules are capable of modulating glucose homeostasis. This modulation is achieved through direct effects on insulin signaling pathways and indirect effects via improving obesity phenotypes. The studies included in this review administered a wide range of milk-derived bioactive compounds, mainly in isolated form. Although most of the studies we found report benefits of carbohydrate- and protein-derived molecules, experiments using lipid-derived bioactives were more equivocal and more research is needed to reach stronger conclusions. It is worth noting that the administered doses of these bioactive molecules may be difficult to achieve through the consumption of milk. Furthermore, milk contains other functional constituents, including BCAAs and exosomal cargos (like microRNAs), whose impact on glucose homeostasis was not elaborated on in this review. Future research should focus on identifying the optimal types and amounts of milk-derived components for preventing and managing metabolic diseases and on understanding the complex interactions between these components and the human body. This will enable the development of targeted dietary recommendations and therapeutic interventions that leverage the full spectrum of benefits offered by milk and its derivatives.

## Figures and Tables

**Table 1:** Results of experimental studies in humans investigating the use of cow’s milk bioactive molecules on glucoregulation-related outcomes.

	Obesity Phenotype		Blood Glucose Parameters	
	Improve	Neutral/Worse	Improve	Neutral/Worse
	Carbohydrate bioactives			
Lactose	--	BMI [38]	GTT [39,40]	FBG, PPG [38]
Galactose	--	--	G-Ra [41,42]	FPG, HOMA-IR [41]
			PPG [41,43]	
			GTT [44,45]	
			FPG [42,46]	
Oligosaccharides—No human studies				
Lipid bioactives				
Milk fat globule membrane	--	BMI [47]	GTT [48]	FBG [47,48,49,50]
				GTT [50]
				HOMA-IR [47]
Polar lipids	WC [51]	BW [52]	--	FBG [51,52]
				HOMA-IR [52]
Even-chain fatty acids—No human studies				
Odd-chain fatty acids	BW, FM [53]	--	FBG, HOMA-IR [53]	--
Trans fatty acids	CLA: BW or BMI, FM (MA [54,55])	--	--	CLA: FBG, HOMA-IR (MA [56])
				RA: FBG [57,58]
				RA: Clamp [58]
Branched-chain fatty acids—No human studies				
Protein bioactives				
Whey hydrolysate	BW [59]	--	FBG [60,61]	HOMA-IR [62]
			GTT [61,63]	
			HbA1c [63]	
			CGMS [60]	
Casein hydrolysate	--	--	FBG [60,61]	
			GTT [61]	
			CGMS [60]	
Peptides—No human studies				

Abbreviations: G-Ra, glucose rate of appearance; BW, body weight; BMI, body mass index; FM, fat mass; FBG, fasting blood glucose; PPG, postprandial glucose; GTT, glucose tolerance test; HOMA-IR, Homeostatic Model Assessment for Insulin Resistance; MA, meta-analysis; CGMS, continuous glucose monitoring system, -- no data. This table provides a summary of relevant literature but does not represent a comprehensive systematic review.

**Table 2:** Results of experimental studies in animal models investigating the use of cow’s milk bioactive molecules on glucoregulation-related outcomes.

	Obesity Phenotype		Blood Glucose		Insulin Signaling	
	Improve	Neutral/Worse	Improve	Neutral/Worse	Improve	Neutral/Worse
	Carbohydrate bioactives					
Lactose	BW [64,65,66]	--	FPG [64,65]	--	--	--
	FM [65]		GTT [67]			
Galactose	BW, FM [68]	BW, FM [69]	FPG [70]	FPG [68]	*Irs2* [68]	--
		BW, FM [68]	Clamp [69]	FPG, HOMA-IR [68]		
			GTT, HOMA-IR [68]			
Oligosaccharides	FM [71]	BW [72]	GTT [73]	FBG [72]	*Pi3k*, *Irs2* [73]	--
	BW [69,71,73,74]		FPG [75]	GTT, HOMA-IR [74]		
			Clamp [69]			
Lipid bioactives						
Milk fat globule membrane	BW [76,77,78,79]	BW [80,81]	FBG [76,77,79,80]	--	*Pi3k*, *Akt* [80]	--
	FM [77]		GTT [76,77,80,81]		PI3K, p-AKT) [77]	
					IRS, AKT [78]	
					AMPK, AKT [79]	
			IP [78]			
Milk polar lipids	BW [82,83,84]	BW [52,85,86,87]	FBG [82,84,88]	FBG [52,86]	(IRS, AKT [85]	--
			GTT [88]	HOMA-IR [52,84,86]		
			HOMA-IR [85]			
Even-chain fatty acids	--	BW [89]	--	FBG, GTT [89]		--
Odd-chain fatty acids	BW [90,91]	BW [92]	FBG [90]	FBG [92]	--	--
			GTT [91,92]			
Trans fatty acids	VA: BW, FM [93]	VA: BW [94,95,96]	VA: FBG, Clamp [94,95]	VA: FBG, HOMA-IR, GTT [96]	VA: IR [95]	--
			VA: FBG, HOMA-IR [93]			
	RA: BW, FM [97]	RA: BW [98,99,100]		RA: FBG, HOMA-IR, ITT [98]	RA: *Ir*, *Irs* [100]	
			RA: FBG, GTT [99]			
			RA: FBG, HOMA-IR, QUICKI, GTT [97]			
			RA: FBG, HOMA-IR) [100]			
		CLA: BW, FM [101]		CLA: FBG, HOMA-IR, R-QUICKI [101]		
Branched-chain fatty acids	--	--	--	--	PDX1, PPAR-γ [102]	--
Protein bioactives						
Whey hydrolysate	--	BW [103,104]	HbA1c [104]	HOMA-IR [105,106]	GLUT-4 [103]	--
			GTT [104,106]	IP [106]		
Casein hydrolysate	BW [107,108,109]	BW [110,111]	GTT [107,109,110,112]	GTT [108]	p-AKT [109,110]	--
			FBG [107,108,111,112]		GLUT-4, AKT, IRS-1 [107]	
			ITT [109]	ITT [110]		
			HbA1c [112]			
			HOMA-IR [111]			
Bioactive peptides	BW [113,114]	BW [115,116]	GTT [113,116,117]	GTT [115]	p-AKT [115]	--
			HOMA-IR [113,114]	HOMA-IR [115]		
			FBG [113,114] ITT [116]	FBG [116]		

BW, body weight; FM, fat mass; FBG, fasting blood (or plasma) glucose; GTT, glucose tolerance test; ITT, insulin tolerance test; HOMA-IR, Homeostatic Model Assessment for Insulin Resistance; IP, insulin production; IR and *Ir*, insulin receptor; IRS and *Irs*, insulin receptor substrate protein and gene; --, no data. This table provides a summary of relevant literature but does not represent a comprehensive systematic review.

## Data Availability

No new data were created or analyzed in this study. Data sharing is not applicable to this article.
